# Validation of the Third Molar Maturation Index (I_3M_) to assess the legal adult age in the Portuguese population

**DOI:** 10.1038/s41598-020-75324-x

**Published:** 2020-10-28

**Authors:** João Albernaz Neves, Nathalie Antunes-Ferreira, Vanessa Machado, João Botelho, Luís Proença, Alexandre Quintas, Ana Sintra Delgado, José João Mendes, Roberto Cameriere

**Affiliations:** 1 Clinical Research Unit (CRU), Centro de Investigação Interdisciplinar Egas Moniz (CiiEM), Egas Moniz, CRL, Monte de Caparica, Portugal; 2 Laboratório de Ciências Forenses E Psicológicas Egas Moniz (LCFPEM), Centro de Investigação Interdisciplinar Egas Moniz (CiiEM), Egas Moniz CRL, Monte de Caparica, Portugal; 3 Laboratory of Biological Anthropology and Human Osteology (LABOH), CRIA/FCSH, Universidade NOVA de Lisboa, Lisbon, Portugal; 4 Evidence-Based Hub, CiiEM, Egas Moniz, CRL, Monte de Caparica, Portugal; 5 Quantitative Methods for Health Research (MQIS), CiiEM, Egas Moniz, CRL, Monte de Caparica, Portugal; 6 Department of Forensic Medicine, University of Sechenov, Moscow, Russia; 7 AgEstimation Project, FOR.MED.LAB, University of Macerata, Macerata, Italy

**Keywords:** Oral anatomy, Dental anthropology, Forensic dentistry

## Abstract

Age estimation is a major step in forensic and legal procedures. Its relevance has been increasing due to growing society issues, such as identification of missing people, crimes against minors or lack of valid identification papers from locals or foreigners. Evaluation of the cut-off value of the Third Molar Maturation Index (I_3M_) = 0.08 for discriminating minors from adults in the Portuguese population. The left lower third molars were analysed by applying a specific cut-off value of 0.08 determined by Cameriere et al. in 2008. A sample of 778 digital panoramic radiographs of a representative Portuguese sample (442 females and 336 males), in the age range of 12–24 years (mean age 17.7 ± 2.98 years in females and 18.1 ± 3.0 years in males), was retrospectively evaluated. I_3M_ decreased as the real age gradually increased in both sexes. The 0.08 cut-off score was valuable in discriminating adults from minors. According to the pooled results, the accuracy, by means of area under the curve, was 92.8% (95% confidence interval (CI) 91.0–94.6%). The proportion of correctly classified subjects (sensitivity) was 90.7% (95% CI 88.7–92.8%) and the specificity was 94.9% (95% CI 93.3–96.4%). The results show that I_3M_ is a valuable method to differentiate minors from adults in the Portuguese population.

## Introduction

Age estimation is a key step in human identification, legal practice and clinical research. Some areas of interest are in the aid of identification of missing people, crimes against minors, adoption procedures, invalid identification documents and mass migration^1–4^. For such reasons, a reliable standardized age estimation tool to differentiate minors from adults is of great interest^1,5^.

Based on research on the distinguishable features of skull bones, symphysis pubis, long bones, hand bones and permanent dentition^2,6–10^, the Study Group on Forensic Age Diagnostics of the German Society of Legal Medicine (AGFAD) presented the criteria for age estimation in lawsuits. Dental examination is highly reliable because dental development is less influenced by both internal and external factors^1,4^. With the exception of the third molars, permanent dentition completed its development between 12 and 14 years of age. Third molars are estimated to develop between the ages of 15.7 and 23.3 years, and because this timeframe intercrosses the legal age of 18, they can serve as a discriminant tool to distinguish adults from minors^1–3,5^.

In Portugal, the age of criminal and legal responsibility is 14 years old. If a crime is committed by an individual between 12 and 16 years old, he will be tried in a juvenile court. In the event of a conviction, the individual may be condemned to serve time in a closed educational center. On the other hand, if the person who commits the crime has between 16 and 18 years of age, may be considered adult and will be judged under general criminal laws. For all purposes, the legal age of adulthood in Portugal is 18 years old^11,12^.

In 2008, Cameriere et al.^13^ proposed the Third Molar Maturation Index (I_3M_) as a predictive tool to discriminate adult age. The I_3M_ relies on the relationship between chronological age and the measures of the open apices of the lower left third molar. The bidimensional widths of the apical and tooth lengths are measured and used to calculate the I_3M_. Then, a cutoff value (0.08) is set to differentiate minors from adults^13^.

Over the last years, I_3M_ was successfully validated worldwide, in Europe^2,13–23^, Africa^1,24–27^, Asia^5,28–31^, America^3,32–36^ and Oceania^37^. Overall, I_3M_ had considerable reliability among all countries, however, to date, this method is not validated for a Portuguese sample. Therefore, investigating if the I_3M_ is suitable for the Portuguese population would be of great interest.

Given the evidence on high population movements in the Portuguese population, contributing to a higher level of diversity than some neighboring populations^38,39^, we aimed to test the validity of the I_3M_ in a Portuguese representative sample, by using panoramic radiographs (PRs).. Secondly, we looked for the influence of sex on its validity. Our null hypothesis was that the I_3M_ has no validity to discriminate adult age in this Portuguese sample.

## Materials and methods

### Source of data and sample size

This retrospective observational study has received approval from the Egas Moniz Ethics Committee (ID 887). Written informed consent was obtained for each participant, during the first appointment at the Egas Moniz Dental Clinic (EMDC), (Almada, Portugal). Regarding participants under 18, a parent and/or legal guardian and this study gave and signed the informed consent. This research was conducted in accordance with the Declaration of Helsinki, as revised in 2013.

The present study follows the Transparent Reporting of a multivariable prediction model for Individual Prognosis Or Diagnosis (TRIPOD) reporting guidelines^40^ for validation of prediction models. This study was conducted on a triple-blind basis with respect to: (1) diagnosis and clinical outcome; (2) data collection; and (3) analysis.

### Participants

A consecutive sample of 1267 digital PRs, taken between September 2017 and January 2020, were considered for this study.

The inclusion criteria were: the presence of the lower third molar; known chronological age between 12 and 24 years old^5,13,36^; and absence of evident bone pathologies or systemic diseases that may affect tooth development. Exclusion criteria included spatial orientation of the third molar that prevents correct measuring, endodontic treatment and/or coronary restoration on the third molar, extensive cavities or abnormal dental anatomy, congenital anomalies and poor-quality or distorted X-rays^41^.

### Outcome, predictors and measurement reproducibility

**Figure 1:**
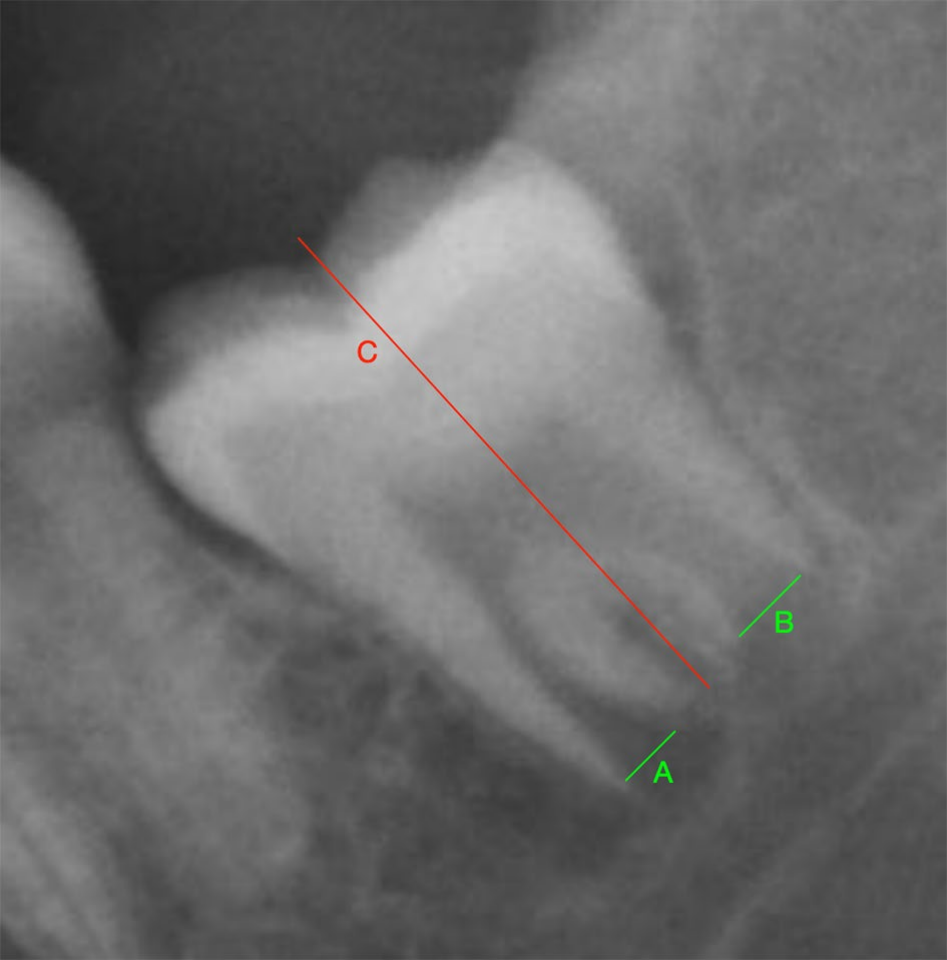
Measurement of the third molar index.

All digital PRs were converted to JPEG documents for the examination with ImageJ image processing software (Graphics Suite X7, Ottawa, Canada) by one trained and calibrated observer (JAN). The I_3M_ index of each evaluated third molar was performed according to the Cameriere et al. method^13^. If the root development of the third molar is complete, then I_3M_ = 0.0. If not, I_3M_ was calculated as the distance between the inner sides of the open apex (A and B) divided by the tooth length (C) (Fig. 1). In case of I_3M_ < 0.08, the individual is classified as 18 years old or older and if I_3M_ ≥ 0.08, the individual as considered a minor.

The Kappa correlation index was used to test the agreement of classification of individuals younger than 18 years, aged 18 years or older. The Intraclass Correlation Coefficient (ICC) was used to analyze the measurements of open apices. Previously, twelve PRs were randomly chosen from the total sample, measured and remeasured one week later by the same researcher (JAN). Kappa index indicated a perfect agreement for the inter-examiner analysis (κ = 1.00). Intraclass correlation coefficient (ICC) showed absolute agreement (ICC = 0.96), according to Landis and Koch^42^.

### Statistical analysis

Data analysis was performed using SPSS Statistics v. 25.0 for Windows (IBM; Armonk, New York, USA). The estimated I_3M_ index result was compared against the actual chronological age to determine the method’s performance in global and according to sex (females and males). For this purpose, contingency tables were used to calculate true positive (TP), true negative (TN), false positive (FP) and false negative (FN) values (Table 1). Then, several performance indicators were determined and detailed in Table 1^43^. Performance measurement was assessed through binary and multiclass area under the curve (AUC), through receiver operating characteristics (ROC) analysis. The correspondent 95% confidence intervals (95% CI) were also determined. Bayes post-test probability (*p*) of being 18 years or older is computed to discriminate between those who are or are not aged 18 years or more. According to Bayes’ theorem, *p* may be written as:

$$p = \frac{Se \,x \,po}{{Se\, x \,po + \left( {1 - Sp} \right)\left( {1 - po} \right)}}$$

In the post-test probability *p*, *p0* defines the probability that a participant is 18 years or older given that he or she is aged between 12 and 24 years, in the target population. In this study, we calculated the probability *p0* as the proportion of participants between 18 and 24 years of age who live in Portugal and those who are aged between 12 and 24 years. This value (*p0*) was considered to be 0.55 (global, males and females) according to the Portuguese National Statistics Institute - Instituto Nacional de Estatística (INE) (https://www.ine.pt/xportal).

**Table 1:** Diagnostic performance indicators used in the comparative analysis.

Sensitivity	TP/(TP + FN)	Proportion positive test results among diseased
Specificity	TN/(TN + FP)	Proportion negative test results among the “healthy”
Accuracy	(TP + TN)/(TP + TN + FP + FN)	Proportion of correctly identified subjects
PPV	TP/(TP + FP)	-
LR +	Sensitivity/(1 − Specifity)	Ratio of the probability a true negative is classified as a true negative
LR-	(1 − Sensitivity)/Specificity	Ratio of the probability a false negative is classified as a true negative
Youden’s index	Sensitivity + Specificity − 1	Measures the performance of a dichotomous diagnostic test
F1 Score	2TP/(2TP + FP + FN)	Harmonic mean of precision and sensitivity
MCC	(TP × TN − FP × FN)/SQRT[(TP + FP)(TP + FN)(TN + FP)(TN + FN)]	Measure of quality of binary classifications

*FN* False Negative, *FP* False Positive; *TN* True Negative; *TP* True Positive; *PPV* Positive Predictive Values; *MCC* Matthews Correlation Coefficient. Adapted from Glas et al. (2003).

## Results

### Participants

A total of 778 digital PRs (336 males and 442 females) met the inclusion criteria, with 489 being excluded due to absence of third molars. The distribution of age and sex is depicted in Table 2. The mean ages of the males and females, aged between 12 and 24 years, were 18.1 ± 3.0 years old and 17.7 ± 3.0, respectively, without statistically significant difference (*p* = 0.078).

**Table 2:** Participants age and sex distribution (n = 778).

Age (years)	Males	Females	Total
12	13	16	29
13	11	22	33
14	10	21	31
15	29	36	65
16	41	64	105
17	51	75	126
18	36	43	79
19	43	38	81
20	28	44	72
21	15	28	43
22	20	22	42
23	27	16	43
24	12	17	29
Total	336	442	778

### Model performance

**Figure 2:**
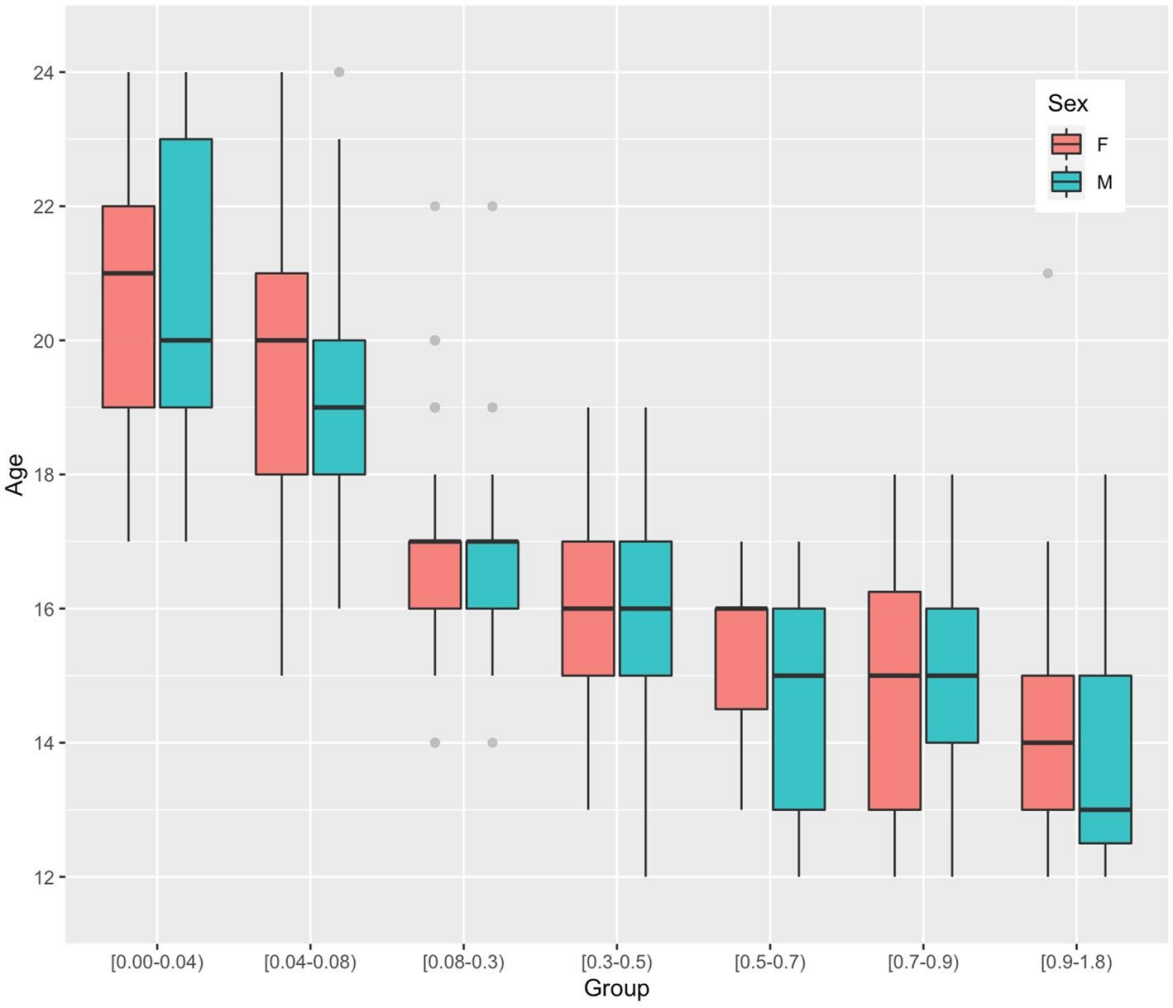
Boxplot of relationship between chronological age and I_3M_ of open apices of lower left third molar, according to females and males.

The estimated age of majority was correlated with the chronological age (p < 0.001). The I_3M_ values decreased as age increased across all age groups in both sexes, showing that the lower third molar mineralization occurred earlier in females than in males (Fig. 2).

Then, pooled data of both sexes as well as separately was analysed (Table 3). The overall results were 92.8% (95% CI 91–94.6%), 90.7% (95% CI 88.7–92.8%) and 94.9% (95% CI 93.3–96.4%) for accuracy, sensitivity and specificity, respectively. Concerning the LR+ and the LR−, their values were as follows: 17.7 (95% CI 12.5–20.3) and 0.10 (95% CI 0.08–0.12). Global post-test probability was 95.6% (95% CI 94.1–97.0%).

**Table 3:** Performance values (in percentage), derived from 2 × 2 contingency tables (with correspondent 95% confidence intervals), of test of age of majority in the Portuguese population.

	Global	Female	Male
Sensitivity	90.7 (88.7–92.8)	87.5 (85.2–89.8)	94.5 (92.9–96.1)
Specificity	94.9 (93.3–96.4)	95.7 (94.3–97.1)	93.5 (91.8–95.3)
Accuracy	92.8 (91.0–94.6)	91.9 (89.9–93.8)	94.0 (92.4–95.7)
Precision	94.6 (93.1–96.2)	94.8 (93.2–96.4)	94.5 (92.9–96.1)
LR+	17.7 (12.5–20.3)	20.5 (15.0–23.3)	14.6 (9.9–17.1)
LR−	0.10 (0.08–0.12)	0.13 (0.11–0.15)	0.06 (0.04–0.08)
AUC	92.8 (90.7–94.9)	91.6 (88.6–94.6)	94.0 (91.1–97.0)
Bayes *p*	95.6 (94.1–97.0)	96.2 (94.8–97.5)	94.7 (93.1–96.3)
Youden’s index	85.6 (83.1–88.1)	83.2 (80.6–85.9)	88.0 (85.7–90.3)
F1 Score	92.7 (90.8–94.5)	91.0 (89.0–93.0)	94.5 (92.9–96.1)
MCC	85.7 (83.2–88.1)	83.8 (81.2–86.4)	88.0 (85.7–90.3)

Males were better classified into adults or minors (94%; 95% CI 92.4–95.7%) than females (91.2%; 95% CI 89.9–93.8%). Specificity was better for females (95.7%; 95% CI 94.3–97.1%) compared to males (93.5%; 95% CI 91.8–95.3%) while sensitivity was better for males (94.9%; 95% CI 93.3–96.4%) compared to females (87.5%; 95% CI 85.2–89.8%). Both estimating post-test probability results were excellent; in males was 94.7% (95% CI 93.1–96.3%) and 96.2% (95% CI 94.2–97.5%) in females. The LR+ and LR− were 20.5 (95% CI 15.0–23.3) and 0.13 (95% CI 0.11–0.15), respectively, in females and 14.6 (95% CI 9.9–17.1) and 0.06 (95% CI 0.04–0.08), respectively, in males.

### Data from articles showing results with cutoff value 0.08 in various European samples

Also, we compared the results of the present study with previous European studies (Tables 4 and 5 present).

**Table 4:** Global performance values (in percentage, with correspondent 95% confidence intervals), of test of age of majority in several European samples.

Author	Country/Region	n	Accuracy	Sensitivity	Specificity	Post-test probability
Cameriere et al*.* (2008)*	Macerata, Italy	906	83.0 (80.6–84.5)	70.0 (67.0–73.0)	98.0 (97.1–98.9)	98.0 (97.0–99.0)
De Luca et al*.* (2014)*	Milan, Italy	397	91.4 (82.8–99.9)	86.6 (88.8–91.1)	95.7 (92.1–98.0)	95.6 (92.0–98.0)
Cameriere et al*.* (2014)*	Rome, Italy	287	88.5 (84.8–92.2)	84.1 (76.7–89.9)	92.5 (87.0–96.2)	90.1 (83.6–95.2)
Rozyło-Kalinowska*et al.* (2017)	Poland	982	86.6 (84.4–88.7)	84.6 (81.9–87.2)	92.0 (88.7–95.3)	96.6 (93.6–99.6)
Spinas et al*.* (2018)	Sardinia, Italy	336	86.0 (82.0–89.0)	82.0 (76.0–86.0)	95.0 (89.0–97.0)	–

*In studies reporting validation of I_3M_ method not reporting 95% Confidence Interval (CI) we calculated 95% CI following Higgins et al. (2011).

**Table 5:** Performance values (in percentage, with correspondent 95% confidence intervals), discriminated by sex, of test of age of majority in several European samples.

Author	Country/Region	n	Accuracy		Sensitivity		Specificity		Post-test probability	
			Male	Female	Male	Female	Male	Female	Male	Female
Galic et al*.* (2014)	Croatia	1416	91.5 (89–93.5)	88.8 (86.3–90.9)	91.2 (88.7–93.1)	84.3 (80.6–87.5	91.9 (88.8–94.3)	95.4 (92.5–97.5)	94.5 (94.3–94.7)	96.5 (95.9–97)
Cameriere et al*.* (2014)	Albania	298	92.5 (89.9–96.2)	87.5 (81.2–90.4)	94.1 (87.6–97.8)	75.4 (68.1–78.8)	90.9 (84.2–94.7)	96.6 (91.1–99.1)	94.4 (88.7–97.3)	97.2 (91.9–99.1)
Zelic et al*.* (2016)	Serbia	589	95.0 (92.0–98.0)	91.0 (87.0–92.0)	96.0 (93.0–98.0)	86.0 (83.0–87.0)	94.0 (90.0–98.0)	98.0 (94.0–99.0)	96.0 (91.0–100)	99.0 (93.0–100)
Gulsahi et al*.* (2016)*	Turkey	293	97.6 (94.9–100)	92.7 (88.7–96.6)	94.6 (88.1–99.8)	85.9 (77.1–92.8)	100	100	100	100
Rozyło-Kalinowska et al*.* (2017)	Poland	982	87.6 (84.8–90.3)	85.3 (82–88.6)	86.2 (82.8–89.6)	82.6 (78.4–86.7)	91.2 (86.7–95.8)	93 (88.3–97.7)	96.3 (92.3–100)	97.0 (92.4–100)
Kelmendi et al*.* (2017)	Kosovo	1221	96.8 (92.6–98.5)	90.9 (87–91.7)	96.2 (92.5–97.8)	82.6 (78.7–83.4)	97.6 (92.9–99.5)	99.1 (95.3–100)	97.5 (90.5–100)	98.9 (92.6–100)
Dogru et al*.* (2017)	Netherlands	360	88.9 (83.3–91.8)	83.3 (77.7–85.8)	84.0 (78.9–86.6)	72.7 (67.6–75)	95.0 (88.7–98.3)	96.3 (90.0–99.0)	95.7 (88.4–100)	96.3 (89–1-100)
Spinas et al*.* (2018)	Sardinia, Italy	336	87.0 (82.0–91.0)	84.0 (78.0–89.0)	85.0 (78.0–91.0)	79.0 (71.0–85.0)	91.0 (82.0–96.0)	100 (92.0–100)	-	-
Tafrount et al*.* (2018)	France	339	91.6 (87.1–90.8)	89.7 (84.2–92.5)	87.1 (80.4–83.4)	81.3 (75–84.5)	95.3 (89.8–98.3)	96.2 (91.3–97)	95.5 (87.7–100)	96.1 (89.1–100)
Antunović et al*.* (2018)	Montenegro	683	93.0 (90.0–96.0)	89.0 (85.0–91.0)	92.0 (88.0–96.0)	82.0 (79.0–94.0)	94.0 (90.0–98.0)	96.0 (93.0–98.0)	96.0 (90.0–100)	97.0 (92.0–100)

*In studies reporting validation of I_3M_ method not reporting 95% Confidence Interval (CI) we calculated 95% CI following Higgins et al. (2011).

## Discussion

The present study is the first to test the validity of I_3M_ in a Portuguese sample. Overall, the null hypothesis was rejected, that is, the I_3M_ is a reliable tool to discriminate adult age in this Portuguese population. Further, I_3M_ was more accurate in male participants. These results have important implications because this tool has potential to be used in legal and criminal settings.

Discriminating minors from adults is important to prevent legal wrongful procedures, specially in cases where valid identification documents are lacking, in order to prevent legal wrongful procedures^4,32^. Notwithstanding, age estimation is challenging, particularly in the differentiation teens from young adults (aged 15 years old to early 20 s), as the physical appearance and characterization are not clearly related of being an adult^1,4^. From 15 years of age, the third molars are only teeth not fully developed, as this clinical circumstance is useful in forensic sciences^44^.

In the Portuguese context, age estimation methods involving dental measures were reported in previous studies^45–50^. Caldas et al. (2011)^46^ have evaluated third molars, however the method and the age range of these participants were not comparable. Thus, to the best of our knowledge, this is the first study that validates the use of the I_3M_ cutoff value of 0.08 in the Portuguese population.

Cameriere et al. (2008)^13^ introduced a new methodology for age estimation based on the ratio between measures of the open apices and height of the third molar. Since then, the I_3M_ has been validated in every continent with very strong results, showing only slight variations among different ethnicities.

The sensibility indicates the I_3M_ ability to correctly identify individuals who are 18 years or older (I_3M_ < 0.08). On the other hand, specificity is the ability to discriminate individuals younger than 18 years old (I_3M_ ≥ 0.08). Santiago et al. (2018)^41^ reported that I_3M_ outperforms in individuals younger than 18 years due to the higher specificity of this tool. This is key in forensic science because if a minor is wrongly processed as an adult it would violate its rights^23,41^.

**Figure 3:**
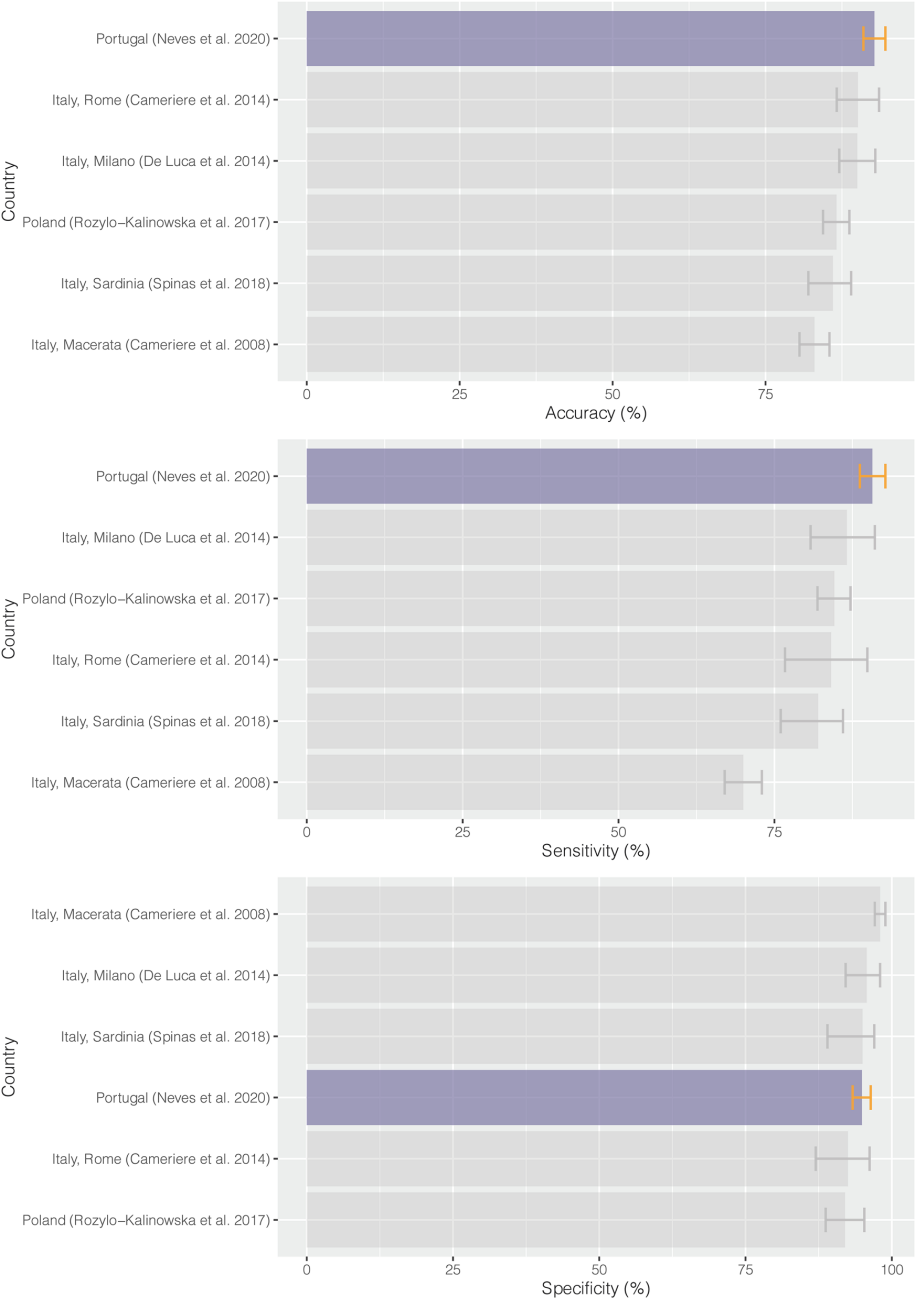
Pooled results from different European populations.

To better integrate the results of our study within the European scenario, we analyzed every European study that have analyzed the validity of I_3M_ (Fig. 3). Our pooled overall results on accuracy (92.8%; 95% CI 91.0–94.6%) outperformed all previous studies that presented pooled data^2,13,17,22,23^ . Both sensitivity and specificity pooled results also surpassed most previous European studies^2,22,23^ except for Cameriere et al.^13^ (98%) and De Luca et al.^17^ (95.7%; 95% CI 92.1–98.0%). The estimated post-test probability of the pooled data (95.6%; 95% CI 94.1–97.0%) showed similar results with previous studies.

**Figure 4:**
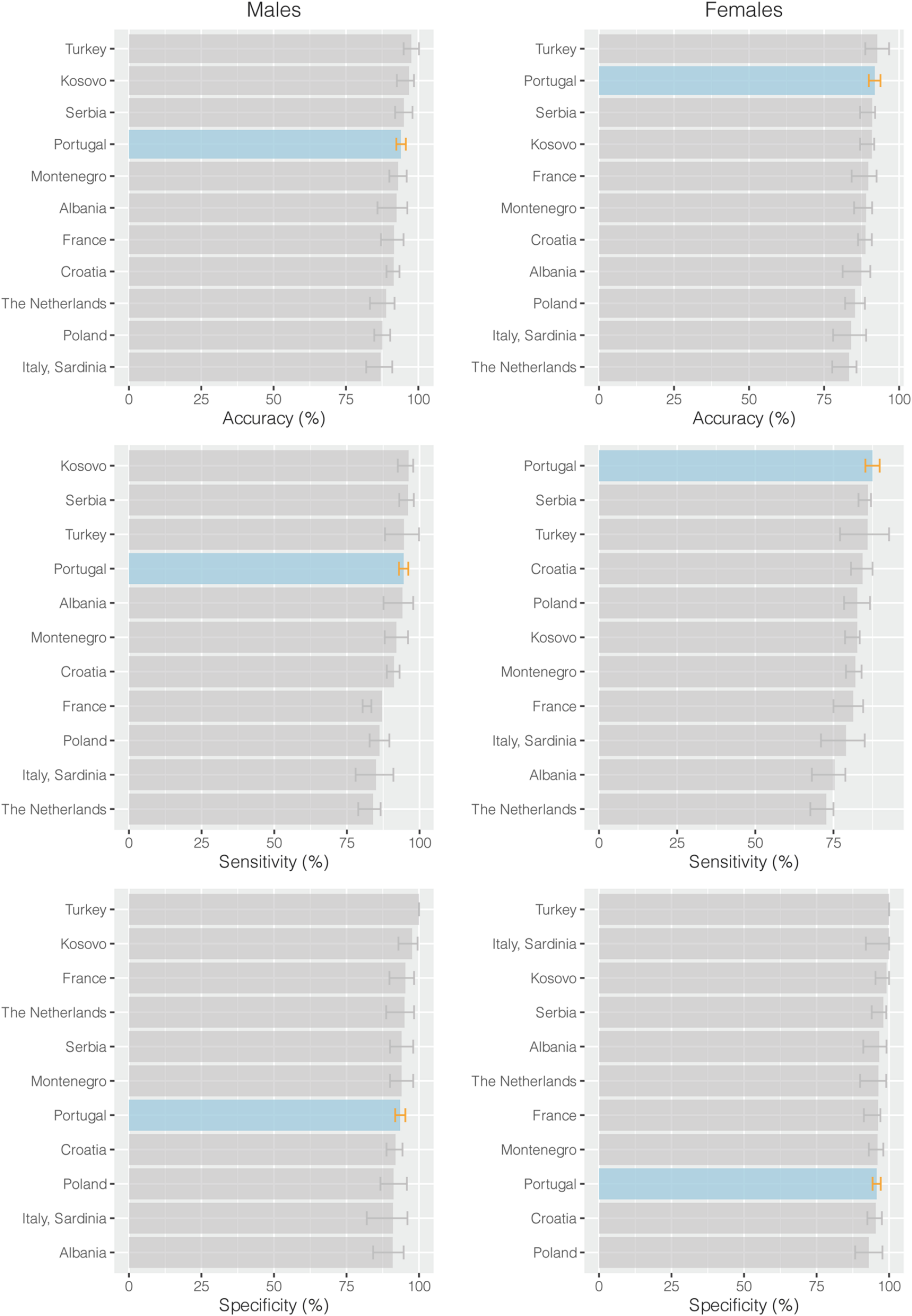
Discriminated results by sex, from different European populations.

When analysing our data regarding males and females (Fig. 4), we demonstrated alike maturation of third molars in both males and females, which is in line with similar studies^14–16,19–23,44,51^. Previous European studies present slightly better accuracy and sensibility in males than in females^14–16,19–23,44,51^. Contrary, females presented better specificity than males^14–16,19–23,44,51^. Therefore, I_3M_ seems to be slightly more accurate in males, although it is equivalent to the female participants in this Portuguese population. The effect of age on I_3M_ performance is proposed to rely on the maturation development according to sex as women tend to develop at a younger ages than men^52^. This maturation difference is suggested to explain the I_3M_ results discrepancies between both sexes, however more studies are needed to fully understand the biological reasons upon this subject^19,41,51^.

Estimated post-test probability of both male (94.7%; 95% CI 93.1–96.3%) and female (96.2%; 95% CI 94.8–97.5%) demonstrated results compatible with other European studies^2,14,15,19–23,44,51^. It is important to refer that Zelic et al.^20^ and Kelmendi et al.^21^ reported results very close to 100% in females, and Gulsahi et al.^19^ reported results of 100% in both sexes, although the sample size was one of the smallest in all European studies (n = 293), which may influence these results.

The likelihood ratio is a practical measure of diagnostic accuracy. The higher the value of LR + , more the test has the capability of establishing the tested condition; an LR + value greater than 10 defines it as a good diagnostic test. In our study, balanced values of LR + and LR − have been achieved. Although I_3M_ presented higher LR + in females (20.5; 95% CI 15–23.3) then males (14.6; 95% CI 9.9–17.1), I_3M_ is an excellent prediction of the probability of majority; and lower LR − (0.06; 95% CI 0.04–0.08) in males than (0.13; 95% CI 0.11–0.15) in females, prove that the test is also an excellent tool at classifying individuals younger than 18 years of age, despite the sex of the individuals^19,23^.

In conclusion, I_3M_ is a suitable method in legal and forensic purposes to identify minors from adults in the Portuguese population. Further, the cut-off value used (0.08) is predictable and useful to discriminating individuals younger than 18 years of age (high specificity in both sexes).

## Supplementary information

Supplementary Information
